# Supplementary material for: pH Distribution along Growing Fungal Hyphae at Microscale
Source: J Fungi (Basel). 2022 Jun 3;8(6):599. doi: 10.3390/jof8060599 (PMC9224906; doi:10.3390/jof8060599)
Supplement: Supplementary file 1 [file jof-08-00599-s001.zip › jof-1746051-supplementary.pdf]

Table S1. Water contact angles of mycelial surfaces of *Coprinopsis cinerea* and *Synechocystis* pH bioreporter cells.

**Organism**	**Water contact angle (degree)**
C. cinerea	128 ± 2
Synechocystis sp. PCC6803_peripHlu	32 ± 2

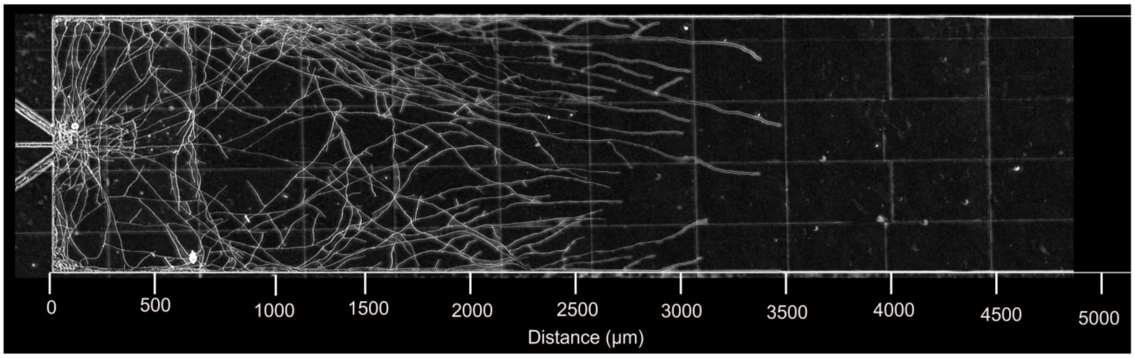

Figure S1. Inverted brightfield micrograph of *Coprinopsis cinerea* hyphal monolayer in the observation chamber before loading of pH bioreporter cells to the microfluidic system. For better visibility of *C. cinerea* hyphae, contrast and brightness of the micrograph were adjusted using ImageJ.

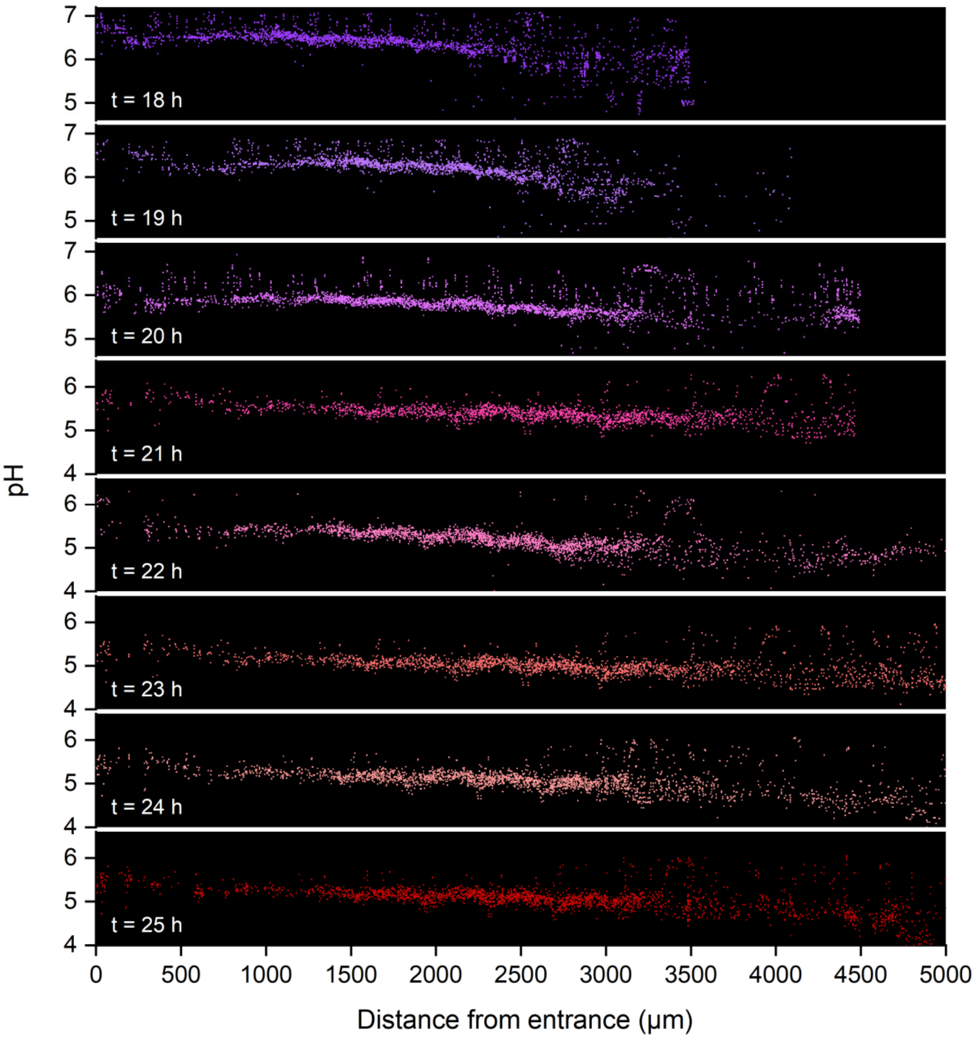

Figure S2. Longitudinal distribution of pH sensed by *Synechocystis* sp. PCC6803 bioreporter cells attached to *C cinerea* hyphae. The x-axis reflects the distance from the entrance to the observation chamber, i.e., where the hyphal tips first enter. pH bioreporter cells reflect a temporally changing and longitudinal pH gradient (≈ 0.8 pH) along the growing *C. cinerea* hyphae.

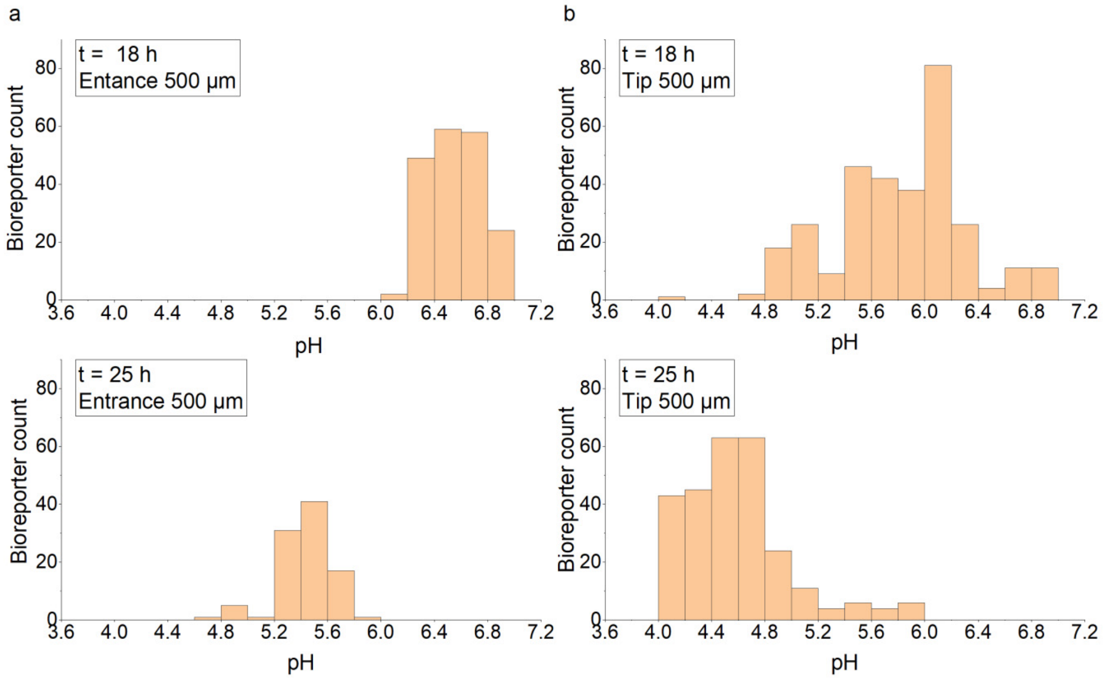

Figure S3. Histogram of distribution of surface pH of (more mature) hyphae near the entrance of the observation chamber and at the hyphal tips of *C. cinerea*. a) pH distribution on hyphal surfaces at 0-500 µm from the start of the observation chamber at t = 18 and 25 h, resp.. b) pH distribution on hyphal surfaces at a distance 500 µm away from the hyphal tips at t = 18 and 25 h, resp.

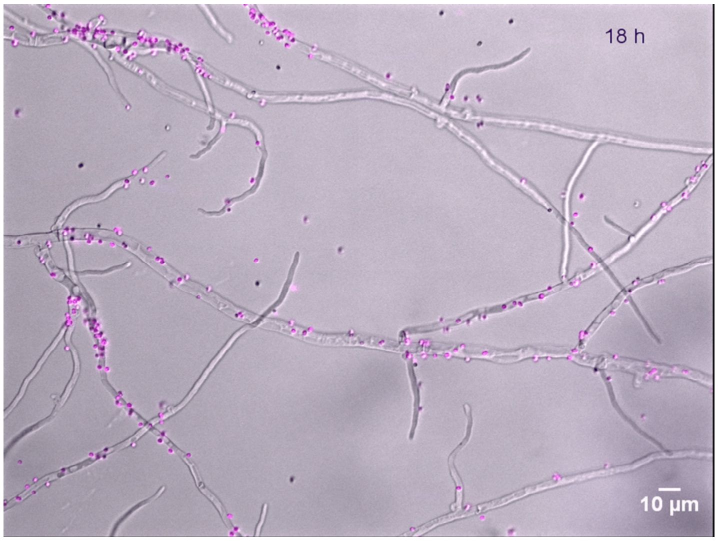

pH Bioreporters distribution on C. cinerea hyphae over time

(Please double click the image to play)

Figure S4. Movement and dispersal **of pH bioreporter *Synechocystis* sp. PCC6803_peripHlu cells along *C. cinerea* hyphae.**
